# Genome-Wide Exploration and Characterization of the *TCP* Gene Family’s Expression Patterns in Response to Abiotic Stresses in Siberian Wildrye (*Elymus sibiricus* L.)

**DOI:** 10.3390/ijms26051925

**Published:** 2025-02-23

**Authors:** Tianqi Liu, Jinghan Peng, Zhixiao Dong, Yingjie Liu, Jiqiang Wu, Yanli Xiong, Changbing Zhang, Lijun Yan, Qingqing Yu, Minghong You, Xiao Ma, Xiong Lei

**Affiliations:** 1 College of Grassland Science and Technology, Sichuan Agricultural University, Chengdu 611130, China; yuji1395659146@126.com (T.L.); m17623220313@163.com (J.P.); dongzhixiao94@126.com (Z.D.); liuyingjieeee@163.com (Y.L.); immortalwjq@outlook.com (J.W.); yanlimaster@126.com (Y.X.); 2 Sichuan Academy of Grassland Science, Chengdu 610097, China; c.b.zhang@126.com (C.Z.); yanlijun456@126.com (L.Y.); yuqinggzu93@126.com (Q.Y.); ymhturf@163.com (M.Y.)

**Keywords:** Siberian wildrye, *TCP* transcription factors, abiotic stress, expression pattern

## Abstract

Siberian wildrye (*Elymus sibiricus* L.), a model *Elymus* Gramineae plant, has high eco-economic value but limited seed and forage yield. *TCP* transcription factors are widely regarded as influencing yield and quality and being crucial for growth and development; still, this gene family in Siberian wildrye remains unexplored. Therefore, this study looked at the Siberian wildrye *TCP* gene family’s reaction to several abiotic stresses, its expression pattern, and its potential evolutionary path. Fifty-four members of the *EsTCP* gene family were discovered. There are two major subfamilies based on the phylogenetic tree: 27 of Class I (*PCF*) and 27 of Class II (12 *CIN*-type and 15 *TB1/CYC*-type). Gene structure, conserved motif, and sequence alignment analyses further validated this classification. *Cis*-elements found in the promoter region of *EsTCPs* are associated with lots of plant hormones and stress-related reactions, covering drought induction and cold tolerance. *EsCYC5*, *EsCYC6*, and *EsCYC7* may regulate tillering and lateral branch development. *EsPCF10*’s relative expression was significant under five stresses. Additionally, eight *EsTCP* genes are potential miR319 targets. These findings highlight the critical significance of the *TCP* gene family in Siberian wildrye, laying the groundwork for understanding the function of the EsTCP protein in abiotic stress studies and high-yield breeding.

## 1. Introduction

As sessile organisms, plants must sustain their growth and development while concurrently adapting to fluctuating environmental conditions. These adaptations in plants involve complex regulatory networks that integrate signals controlling fundamental biological processes such as cell division, differentiation, and organ formation, as well as responses to external stresses such as drought, salinity, temperature extremes, and pathogen attack. Transcription factors (TFs) are central to these regulatory networks, acting as molecular switches that regulate gene expression and fine-tune both developmental programs and stress responses. TFs with dual functions—promoting growth and mediating stress tolerance—are particularly important, as they help plants optimize growth while coping with environmental stress.

The *TCP* (*Teosinte branched1*, *Cycloidea*, and *Proliferating cell factor*) family is a highly conserved set of plant-specific regulators that play critical roles in growth, development, and environmental response [1]. Three paradigmatic genes serve as the basis for the *TCP* family’s nomenclature: rice *Proliferating Cytokinin Factors 1/2* (*PCF1/2*); *CYCLOIDEA* (*CYC*), which controls bilateral floral symmetry in snapdragon; and *TEOSINTE BRANCHED1* (*TB1*), which controls apical dominance in maize [2]. *TCP* transcription factors are central regulators of several developmental processes, including leaf morphogenesis, axillary meristem development, floral organ formation, and branching of meristematic tissues [3,4,5]. Apart from their roles in development, TCP proteins also integrate hormone signaling networks that include gibberellin, auxin, abscisic acid, and jasmonic acid [6]. This allows them to control how plants react to both biotic and abiotic stress. By coordinating growth and stress response networks, TCP proteins exhibit an integral role in plant adaptation and survival. This versatility makes the *TCP* family a key component in understanding the molecular mechanisms of plant development and environmental resilience. Based on the structure of the TCP domain, the *TCP* gene family is separated into two main subfamilies: Class I (*PCF* or *TCP-P*) and Class II (*TCP-C*) [7]. The most notable feature of Class I *TCP*s is their shorter TCP domain, which lacks four amino acids in comparison to Class II members [2,8,9]. These *PCF* transcription factors [10] primarily regulate cell proliferation and elongation, promoting plant growth and development through the modulation of hormonal signaling pathways, including gibberellins, auxins, cytokinins, and abscisic acid. Class II *TCPs* are functionally diverse and divided into two subclades: *CIN*, which governs lateral organ development, and *TB1/CYC* [11,12], which controls apical dominance, stem branching, and axillary meristem activity. Interestingly, many Class II members also feature an additional arginine-rich motif known as the R domain [13], although its specific function remains unclear. The differentiation between these two classes reflects the evolutionary diversification of TCP proteins and their broad roles in regulating plant adaptability and development. The importance of *TCP* genes is well-documented across species. As an illustration, the *TB1* gene in maize regulates apical dominance and tillering, while its homologs in Arabidopsis (*AtTCP18/BRC1* and *AtTCP12/BRC2*) and rice similarly repress lateral branching [14,15]. *TCP* genes are involved in the responses of rye (*Secale cereale* L.) to cold and drought stress, and the *TB1* homolog *ScTCP9* interacts with *ScFT* to control flowering time [16]. Similar regulatory roles have been shown in other species, such as orchardgrass (*Dactylis glomerata* L.), where many *TCP* genes are upregulated during stress, implying their involvement in the regulation of stress responses [17]. Likewise [18], the conserved function of *TCP* genes in controlling stem and meristem development is shown by the tillering-associated *TCP* genes found in Foxtail millet (*Setaria italica* L.) and its near relative Green foxtail (*Setaria viridis* L.).

Siberian wildrye (*Elymus sibiricus* L.) is a self-pollinating, heterotetraploid perennial forage grass with a genome composition of StStHH (2n = 4x = 28) [19,20]. It belongs to the genus *Elymus*, subfamily Triticeae, and family Poaceae and holds significant ecological and agricultural value. The species is widely distributed across Eurasia, thriving in regions such as the Tibetan Plateau, northeastern China, Inner Mongolia, and Xinjiang. Its key traits—robust adaptability, cold and drought tolerance, salinity resistance, and high nutritive value—make it invaluable for rangeland restoration, rotational grazing, and forage production. Nevertheless, the short growing season and erratic precipitation in semi-arid regions such as the Tibetan Plateau limit its seed production and biomass. Tiller traits directly affect plant structure and yield, and improving tillering traits has become a key focus of genetic improvement and high-yield breeding in Siberian wildrye. The regulation of tillering involves complex interactions between gene expression and hormone signaling pathways, which are usually mediated by specific families of transcription factors. Among these, the *TCP* gene family is of special significance for balancing vegetative and reproductive growth, regulating hormone pathways, and modifying responses to both abiotic and biotic stresses [21]. The *TCP* gene family is essential for controlling a host of plant life, although it is unknown exactly how it affects Siberian wildrye.

In conclusion, despite the fact that the *TCP* gene family has been thoroughly investigated in numerous plant species, more research is still required to fully identify and characterize its functions in Siberian wildrye. The purpose of this study was to completely identify the *TCP* gene family in Siberian wildrye at the genome level, evaluating its evolutionary relationships, gene structural properties, and expression profiles under a variety of environmental situations. The study sheds light on the molecular mechanisms underpinning fodder adaptation by identifying the critical role of *TCP* genes in controlling growth, development, and stress responses in Siberian wildrye. Additionally, this study established a strong basis for elucidating the role of the *TCP* gene in Siberian wildrye and kindred species. Furthermore, this understanding has broad potential value for enhancing the productivity and tolerance characteristics of different forages and creates a new avenue for broad use in the field of feed crop enhancement.

## 2. Results

### 2.1. Identification of TCP Genes in Siberian Wildrye

**Figure 1:**
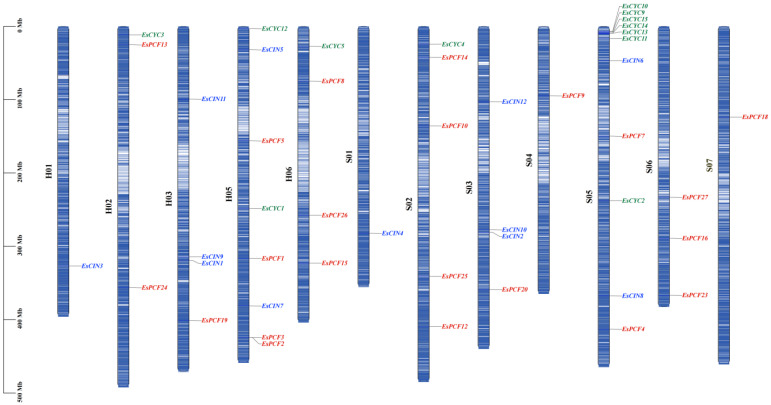
The *TCP* gene family members’ chromosomal locations in Siberian wildrye. An increase in gene density is indicated by the chromosomes’ color changing from blue to white. Gene positions are shown on the right side of the vertical bar graph, while chromosomal numbers are shown in black on the left. The first class of *EsPCF* genes is shown in red, and the second class of *EsCIN* and *EsCYC* genes are shown in blue and green, respectively.

We sought *TCP* members in the Siberian wildrye genome using HMMER 3.0 and the HMM file of the *TCP* transcription factor (PF03634) from the Pfam database [22,23,24]. The CDD database was used to assess the TCP domain, and blast comparison was used to remove any redundant genes [25,26,27]. Eventually, 54 *TCP* candidate members were chosen. Then, the 54 potential *TCP* members that had been found were categorized based on their conserved motifs, TCP domain presence, and similarity to the *TCP* genes that were being studied. The physical and chemical characteristics of these proteins were determined to be hydrophilic and unstable (Table S1). The genomic length of 54 *EsTCPs* varied from 396 bp to 1791 bp, corresponding to *EsPCF12* and *EsCIN10*; their pI values showed significantly (pI value 4.90~11.71). Except for a few proteins located in the chloroplast (EsPCF7, EsPCF13, EsCIN10, EsCYC4, EsCYC11) and mitochondrial matrix (EsPCF12, EsPCF22, EsCYC3), most of the *TCP* members are predicted to be located in the nuclear. It can be seen that eight *TCP* members are unplaced in Siberian wildrye, and the other members are distributed on the St (S01, S02, S03, S04, S05, S06, S07) and H (H01, H02, H03, H05, H06) chromosomes, respectively (Table S1, Figure 1).

For the multiple sequence alignment of putative TCP proteins, we used Clustal Omega (https://www.ebi.ac.uk/jdispatcher/msa/clustalo?outfmt=fa, accessed on 18 February 2025) on the EBI platform [28]. Further analysis confirmed that the EsTCP proteins can be categorized into two distinct classes, as illustrated in the figure below, consistent with observations in other plant species. Twenty-seven members (EsPCF1 to EsPCF27) were in Class I, while the rest were in Class II, which included 12 CIN-type (EsCIN1 to EsCIN12) and 15 TB1/CYC-type (EsCYC1 to EsCYC15).

### 2.2. Phylogenetic Analysis of the TCP Proteins

**Figure 2:**
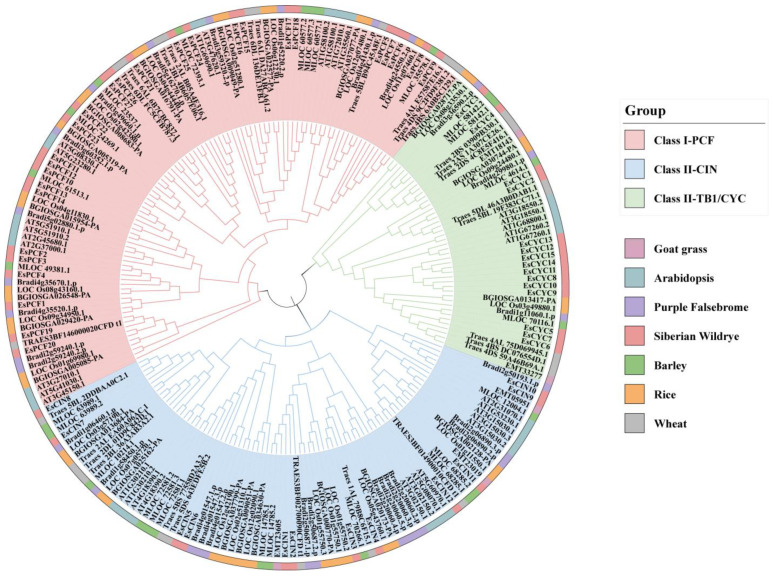
A phylogenetic tree of TCP proteins from Goat grass, Arabidopsis, Purle Falsebrome, Siberian wildrye, barley, rice, and wheat. TCPs were split into three groups (PCF, CIN, and TB1/CYC) based on protein sequence clustering. The proteins from Goat grass, Arabidopsis, Purle Falsebrome, Siberian wildrye, barley, rice, and wheat are presented in grayish purple, baby blue, purple, pink, blue, green, orange, and gray, respectively.

A maximum likelihood phylogenetic tree was constructed using 218 protein sequences in an attempt to reveal the evolutionary relationships of TCP proteins in Arabidopsis (*Arabidopsis thaliana* L.), Goat grass (*Aegilops tauschii* L.), rice (*Oryza sativa* L.), barley (*Hordeum vulgare* L.), Purple Falsebrome (*Brachypodium distachyon* L.), wheat (*Triticum aestivum* L.), and Siberian wildrye. Based on the subtle variations in base amino acid sequences and motifs within their conserved domains, as well as the branching pattern in the phylogenetic tree, we have discovered that all TCP proteins can be categorized into three distinct clades. Figure 2 depicts a phylogenetic evolutionary tree that clearly divides TCP proteins into three different branches. For instance, the CIN and TB1/CYC subbranches together comprise Class II, often known as TCP-C [8]. This also confirmed previous studies in which TCP proteins are divided into two major categories: Class I and Class II. The second category is further divided into two subclasses, CIN and TB1/CYC. Half of the 54 TCP proteins in the Siberian wildrye belong to PCF, and the first class has the largest number of members, which also shows this result in the multi-species phylogenetic tree. The evolution of TCP proteins in rice and Arabidopsis is strongly tied to the bulk of EsTCPs, despite the fact that Arabidopsis is the sole dicotyledonous plant on the evolutionary tree. This suggests that there is no meaningful particular link between species and that the homology of TCP proteins primarily resides in amino acid sequences.

### 2.3. Multiple Sequence Alignment and Studies of the Gene Structure and Conserved Motif of EsTCPs

**Figure 3:**
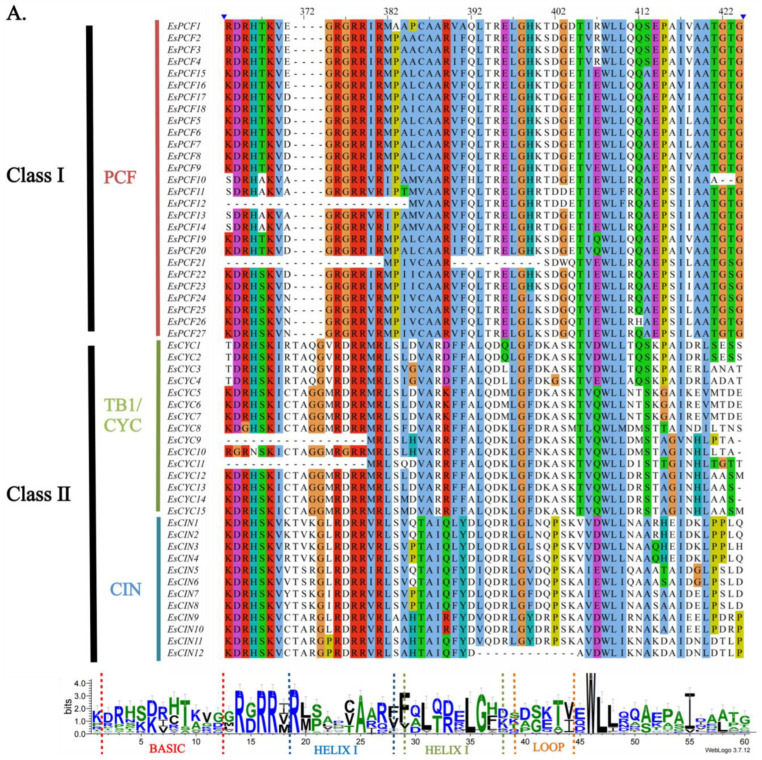

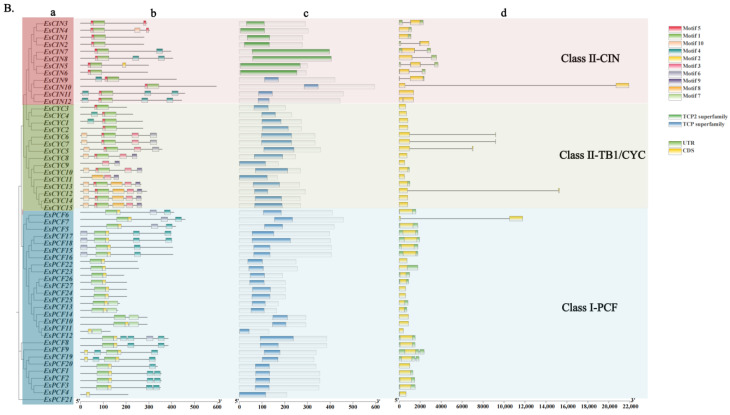
(**A**) Multiple sequence alignment and conservative domain of TCP protein in Siberian wildrye. The blue triangle indicates conserved regions of interest, and the bases within these conserved regions of the EsTCP protein sequence are highlighted in corresponding colors. (**B**) Phylogenetic tree, motif analysis, conservative domains, and gene structure of EsTCPs: (**a**) Phylogenetic tree analysis of the EsTCP proteins. (**b**) Motif composition of EsTCP proteins. (**c**) Conservative domains of EsTCP proteins. (**d**) Gene structure of the *EsTCP*s.

A comprehensive investigation of the *EsTCP* family’s gene structure and domains shed light on the *TCP* family’s evolutionary history in Siberian wildrye. According to the results of multiple alignments, all TCP proteins have a conserved TCP structural domain. This domain comprises a basic portion of 59 amino acid residues and a helix-loop-helix (bHLH) domain, which is critical for DNA binding and protein interactions [2]. This is consistent with prior research [29,30,31], which showed that members of the *TCP* family of Siberian wildrye might be made up of two classes depending on the number of amino acids (Figure 3A). This grouping result correlates with that of the phylogenetic tree grouping.

As depicted in Figure 3B, certain genes pertain to the *TCP2* superfamily (*EsCIN1*-*EsCIN8*). These genes possess similar structures, and the functions of their expression products are potentially diverse. Apart from the fact that some genes do not contain introns (such as *EsPCF17*, *EsPCF18*, *EsPCF8*, *EsPCF9*, etc.), all the other genes in this set contain 0 to 1 intron. To gain a more in-depth knowledge of the structural features of this protein, we used the MEME tools (https://meme-suite.org/meme/tools/meme, accessed on accessed on 18 February 2025) [32,33] to detect ten conserved sequence patterns in the EsTCP protein and look into how these patterns appear in the TCP protein (Figure 3B and Figure S1). The related results show that the ten most conserved motifs observed contain 11 to 50 amino acids. The bulk of these genes contain motif 1, indicating that the gene family in Siberian wildrye is substantially conserved. All Class I TCP proteins in Siberian wildrye have motif 1 and motif 2, while both Class II EsTCPs contain motif 1 and motif 5. Clearly, related proteins have similar patterns, implying that the *EsTCP* gene family performs similar activities and that few conserved motifs were lost during the evolutionary process. Additionally, genes positioned on the identical branch of the evolutionary tree share comparable gene structures and quantities of motifs. The coding region length and position of each member in the same classification are similar, while the differences between groups are greater.

### 2.4. Duplication and Collinearity Analysis of TCP Genes

**Figure 4:**
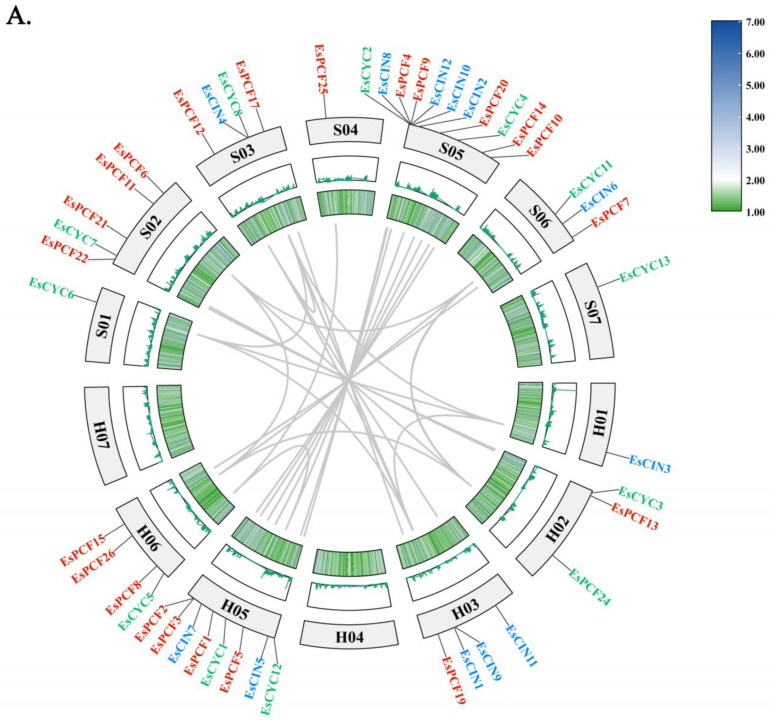

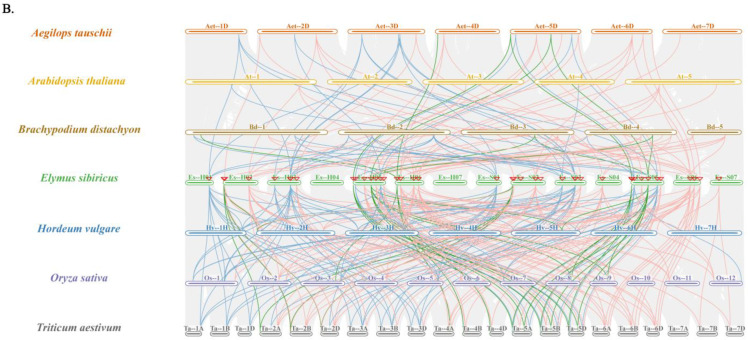
(**A**) Gene duplications of the *TCP* gene family in Siberian wildrye. The gray lines in the diagram represent *EsTCP*’*s* duplicated gene pairs, while black letters denote chromosome numbers. Class I *EsPCF*, Class II *EsCIN*, and *EsCYC* are marked in red, blue, and green, respectively. The density of genomic genes in Siberian wildrye increased from dark green and white to dark blue. (**B**) Analysis of synteny of *TCP* genes in Siberian wildrye and six other representative plants. The collinearity between the genomes of Siberian wildrye and other species is represented by the gray background line. The red triangle represents the equivalent *EsTCP* genes on the genome chromosome, and the Latin abbreviation of a species here denotes its location on its genome. The *EsTCP* genes of the three groups (*PCF*, *CIN*, and *CYC/TB1*) are represented by the pink, blue, and green lines, respectively.

*EsTCP* gene duplication occurrences were examined using One Step MCScanX-Super Fast [34]. Combined with Figure 1, it is found that there are two tandem repeat genes, *EsPCF2* and *EsPCF3*, in Siberian wildrye (Figure 4A). Twenty-nine genes are of fragment duplication and distributed in two major categories, among which there are more duplication events in *EsPCF* genes. In addition, we estimated the sub-conformational rates of distinct meanings (Ka) and synonyms (Ks), as well as the Ka/Ks ratio (Table S2), to reflect the evolution of TCP protein-coding sequences. According to the findings, all 29 pairs of duplicated genes had Ka/KS values smaller than 1, indicating that purifying selection has significantly impacted these genes. These associated duplication genes anticipate the same function of *cis*-acting elements in the promoter region, particularly in meristem expression and plant hormone response (Figure S2, Table S4), implying that they are functionally equivalent. For instance, we did a thorough investigation of functional similarities within the *TCP* gene family and discovered that *EsPCF24* and *EsPCF27* show resilient, functional conservation in plant stress response and developmental control (Figure S3 and in below). Both genes showed similar expression patterns in response to exogenous plant hormones (ABA, IAA, and MeJA) in terms of hormone responsiveness. In particular, *EsPCF24* and *EsPCF27* were usually reduced at most time points in other treatments, but they were markedly upregulated in leaves at 3 and 12 h after ABA therapy. These genes also showed synergistic expression in response to abiotic stress under low-temperature and drought circumstances. They were especially significantly upregulated during three hours of drought stress, suggesting that they may play a role in the plant’s drought resistance mechanism. Furthermore, tissue-specific expression analysis demonstrated that *EsPCF24* and *EsPCF27* remained highly expressed during root development, implying that these homologous genes may play comparable functions in regulating root growth.

To explore the evolutionary relationship of the Siberian wildrye *TCP* gene with other species, the gene sequences of the dicotyledonous model plant Arabidopsis and representative plants of the Poaceae family, such as Goat grass, Purle Falsebrome, barley, wheat, and rice, were analyzed (Figure 4B). While almost fifty pairs of analogous *EsTCP* genes were found in rice, barley, Goat grass, and Purle Falsebrome, just ten pairings were revealed in Arabidopsis (Figure 4B, Table S3). One hundred and thirty-six pairs of comparable genes detected in wheat provide substantial support for the high degree of consistency in the evolutionary history and functional characteristics of *TCP* genes in Siberian wildrye and wheat (Table S3).

### 2.5. miR319 Target Sites Prediction of EsTCPs

**Figure 5:**
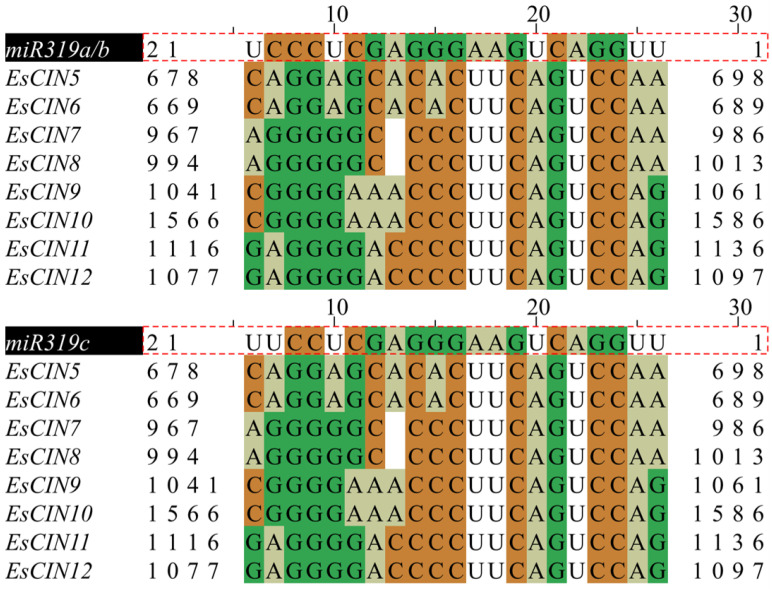
Alignment in potential miR319 target regions. The sequence enclosed by black shadows and red dashed lines represents the name and sequence of miR319, while the conserved bases in the predicted *EsTCPs* target gene sequence are highlighted in corresponding colors.

It has been discovered that miR319 can affect the *TCP* gene post-transcriptionally. *AtTCP2*, *AtTCP3*, *AtTCP4*, *AtTCP10*, and *AtTCP24*, as well as Class II *TCP* homologs in other plants, are thought to be regulated by miR319 [4], which has been preserved throughout evolution. In rice, miR319 targets five *TCP* genes (*OsPCF5*, *OsPCF6*, *OsPCF7*, *OsPCF8*, and *OsTCP21*) [35,36]. Similarly, eight *EsTCP* genes include miR319 binding sites in the CDS, and these miR319-targeting *EsTCPs* belong to the *CIN* subclass (Figure 5). Putative recognition sites for miR319 have been identified for *EsCIN5*-*EsCIN12* in Siberian wildrye, where both *EsCIN7* and *EsCIN12* are highly expressed in roots, but their differences in response to different abiotic stresses lead to presumed specificity of their expression and response (Figure S3 and in blew). For example, when exogenous abscisic acid is applied to leaves, *EsCIN7* expression is inhibited, but *EsCIN12* expression seems to be stimulated and increased. On the other hand, leaves treated with polyethylene glycol (PEG) showed a substantial increase in *EsCIN7* expression (Figure S3), suggesting that this gene may be involved in drought stress response. Additionally, after IAA treatment, *EsCIN12* showed limited expression in either the leaves or the roots, indicating that auxin signaling pathways may be adversely regulating its expression (Figure S3 and in blew). The divergent expression patterns of *EsCIN7* and *EsCIN12*, both anticipated miR319 targets, demonstrate the functional variety of these genes.

### 2.6. Examination of Cis-Acting Elements Within the Promoters of the EsTCP Genes

**Figure 6:**
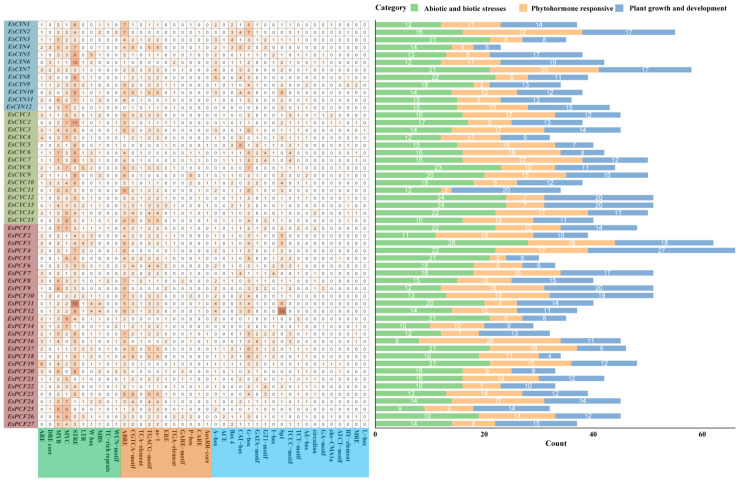
Regulatory *cis*-elements positioned on the promoters of *EsTCP* genes. On the left side of the figure, the distinct elements of each *EsTCP* gene are individually calculated, with the background color of the element annotations progressively darkening as their values increase. On the right, all components are classified into three broad categories: abiotic and biotic stresses, phytohormone responsive, and plant growth and development, which are color-coded in green, orange, and blue, respectively.

We utilized PlantCARE (https://bioinformatics.psb.ugent.be/webtools/plantcare/html/, accessed on 18 February 2025) predicted *cis*-acting elements to describe the expression of each *EsTCP* gene after extracting the upstream 2000 bp sequences of each gene promoter region from the genome of Siberian wildrye [37,38]. A total of 40 *cis*-acting elements that are linked to hormone response, abiotic and biological stress, and plant growth and development were analyzed (Figure 6). The most *cis*-regulatory elements are found in *EsPCF4*, whilst the fewest are found in *EsCIN4*. Additionally, it is anticipated that most *EsTCP* gene promoters contain components linked to drought, methyl jasmonate, auxin, abscisic acid, and low-temperature responses, demonstrating the *TCP* gene family’s capacity to adapt to abiotic stress. It was also found that several genes have *cis*-acting regulatory regions involved in meristem expression (Figure S2, Table S4). These *cis*-acting elements serve an essential function in the *EsTCPs* gene expression regulation network, integrating numerous signal transduction pathways to ensure accurate and spatiotemporal-specific gene expression in complicated biological processes.

### 2.7. Expression of TCP Genes Was Detected by RT-qPCR

**Figure 7:**
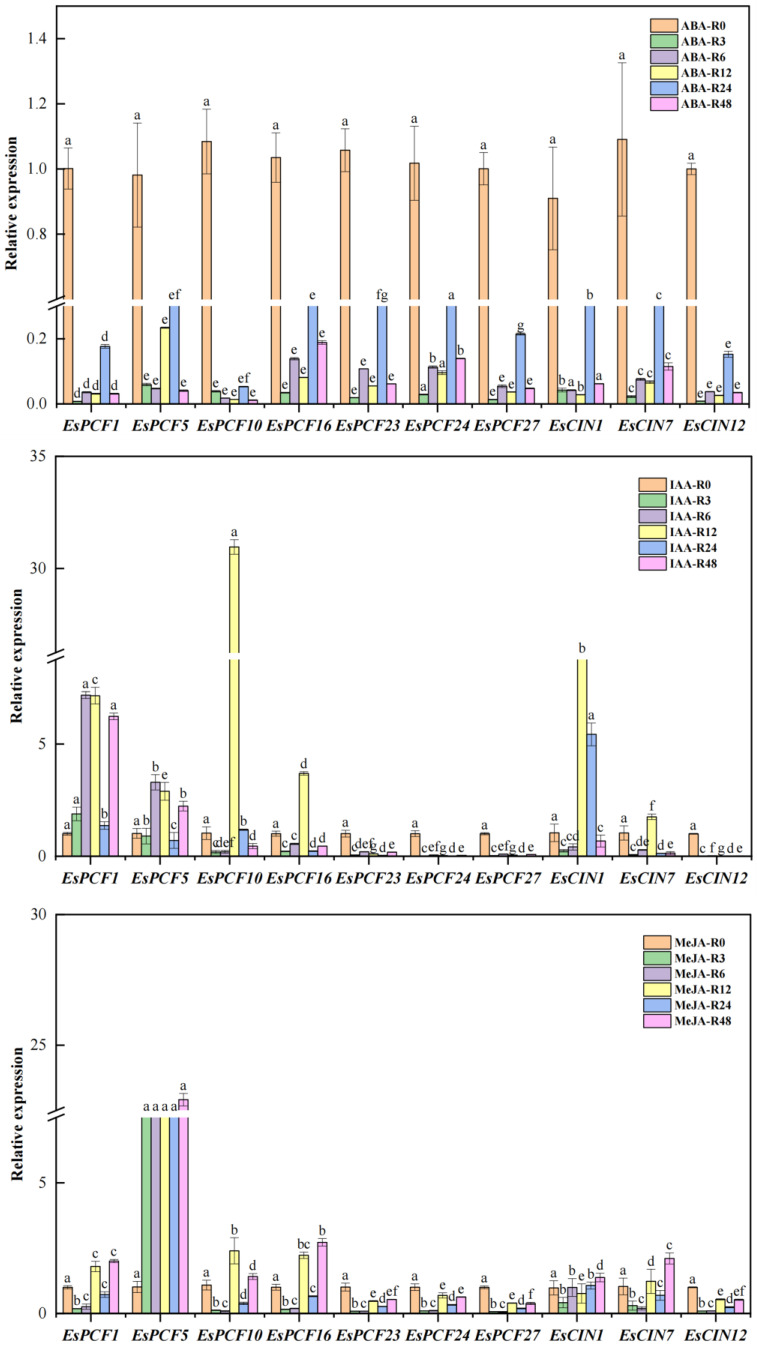

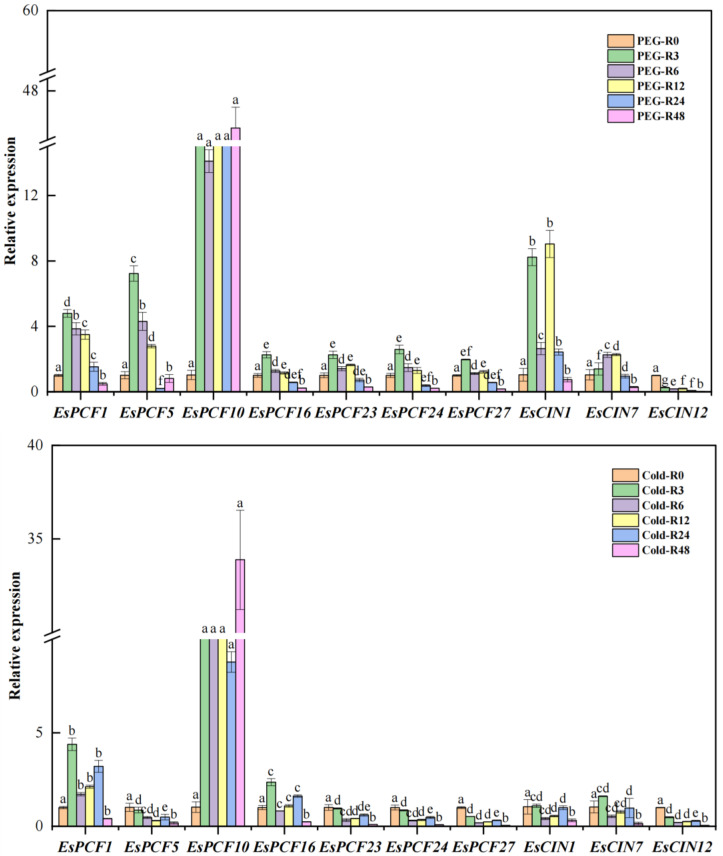
Ten *TCP* genes’ expression levels in Siberian wildrye roots at 0, 3, 6, 12, 24, and 48 h under five different stress conditions (ABA, IAA, MeJA, PEG, Cold −4 °C). Significance levels in the figure are denoted by lowercase letters (a–g), with a 95% confidence interval (*p* < 0.05).

**Figure 8:**
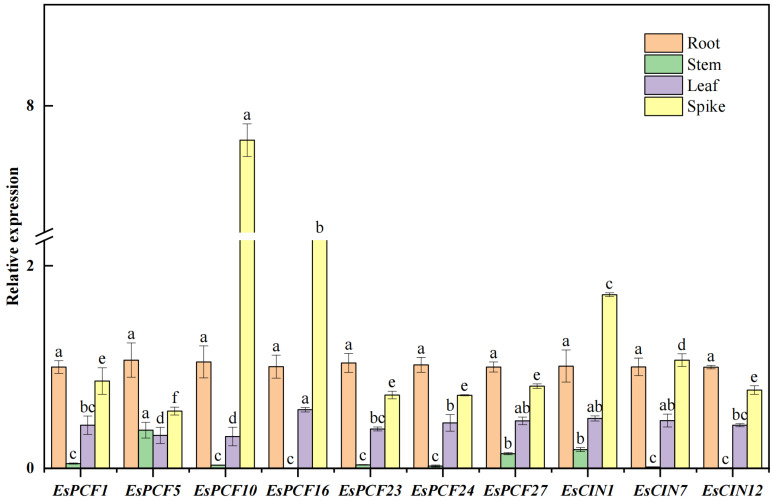
Relative expression of *EsTCP* genes in diverse tissues. Under normal growth conditions, the expression levels of 10 *EsTCP* genes in roots, stems, leaves, and spikes of Siberian wildrye were respectively. Significance levels in the figure are denoted by lowercase letters (a–f), with a 95% confidence interval (*p*<0.05).

To investigate the expression features of the *EsTCP* gene family under various abiotic stress settings, ten representative *EsTCP* genes from each major branch were screened for Quantitative Real-time PCR (RT-qPCR) expression pattern detection via phylogenetic and evolutionary analysis. To avoid repetitive investigations, homologous genes with high sequence similarity in smaller branches were removed to guarantee that the selected genes accurately represented the variety of the *EsTCP* gene family. Also, prior examinations of promoter *cis*-acting elements suggested potential functional associations between these picked genes and other biological functions or stress reactions (Figure 6). Under five abiotic stress settings, the genes in the current research displayed especially varied expression patterns, as illustrated in Figure 7, Figure 8, and Figure S3. Following ABA treatment, each gene demonstrated distinct tissue specificity in its expression, with the expression level at 0 h (prior to stress treatment) being relatively high. This suggests that ABA suppressed the expression of the *TCP* gene in roots. Under IAA treatment, gene expression levels were generally low, and most *TCP* genes had the highest relative expression levels at the 12th hour of stress (*EsPCF10*, *EsPCF16*). The relative expression initially increased and then reduced as the duration of IAA treatment increased. In contrast to *EsPCF10* and *EsPCF16*, *EsPCF5* exhibited weaker expression in MeJA-treated leaves while demonstrating significantly upregulated expression in root tissues (*p* < 0.05). Compared with other genes, the relative expression of the *EsPCF10* gene was more prominent under PEG-simulated drought stress and low-temperature stress. Our work unequivocally demonstrates that *TCP* genes are necessary for plants to withstand abiotic stress.

In addition, we also collected the new root tips, young leaves, new stems, and young ears of Siberian wildrye under normal growth conditions and tested the gene expression of the same 10 genes in different tissue parts (Figure 8). *EsCIN1* and *EsCIN7* exhibit the highest expression levels in leaves, whereas *EsPCF10* and *EsPCF16* show peak expression in young ears. These findings clearly demonstrated that the *EsTCP* gene exhibits tissue-specific expression and that each member gene may have separate regulation mechanisms and expression traits. Simultaneously, it was discovered that the *TCP* transcription factor family is extensively involved in and modulates the biological processes across multiple vital developmental stages and that it plays a crucial regulatory role in the Siberian wildrye’s entire growth and development cycle.

### 2.8. Three-Dimensional Structure and Protein Interaction Network Prediction of EsTCP Proteins

To predict the three-dimensional structure of the EsTCP protein, we applied the SWISS-MODEL program (https://swissmodel.expasy.org/interactive, accessed on 18 February 2025) in conjunction with the homology modeling method [39]. Structures with a sequence identity of 30% or greater to the target sequence were identified as feasible templates (Figure S4), and the TCP protein structure obtained from the Protein Data Bank (PDB) was chosen as the template. The predicted model consistency for all EsTCP proteins ranged from 44.62% to 96.68%, and the corresponding GMQE (Global Model Quality Estimation) values varied from 0.14 to 0.63 (Table S5). It can be concluded that the model exhibits a high degree of stereochemical rationality. Subsequently, these models were predicted to be reliable and were further subjected to quality assessment through the SAVES v6.1 server. The server can offer five evaluation results: PROCHECK, WHATCHECK, ERRAT, Verify-3D, and PROVE, among which the indication of passing in three of them implies that the model is available [40,41,42,43,44]. The results showed that 57.1429 and 100, respectively, were the lowest and highest ERRAT scores (Table S5). Generally, a quality score of >85 is regarded as good, and the crystal could reach 95. Twenty-two of the templates are of acceptable quality, hinting that the remaining ones may require further revision. Then, the EsCYC8 protein is anticipated to form a homologous dimer, albeit its species origin is unknown, and it is thought to operate as the transcription factor *TCP10*. In *Phyllostachys edulis*, *PeTCP10* has been shown to mediate drought and abscisic acid (ABA) responses [45]. Based on these results, we hypothesize that the *EsCYC8* gene in Siberian wildrye responds comparably to drought and ABA stimuli. Furthermore, the predicted EsTCP homologous proteins are mostly distributed in rice, wheat, and maize and have also been predicted in *Oryza meyeriana*, Goat grass, Purple Falsebrome, and barley. The evolutionary relationship between Siberian wildrye and maize, wheat, rice, Goat grass, barley, and other crops was established with three-dimensional structure prediction, revealing that *TCP* transcription factors are substantially conserved throughout the evolutionary process.

A further examination of the protein interaction network was carried out in order to look into the interactions within EsTCP proteins. On the STRING website (https://cn.string-db.org/cgi/input?sessionId=bFSnR2S3L0Ea&input_page_show_search=on, accessed on 18 February 2025) [46], we submitted all the TCP protein sequences of Siberian wildrye, among which 29 sequences were predicted to be homologous proteins of wheat. Taking the data of the wheat genome as a reference, we obtained the protein interaction network of EsTCP. Numerous Class I PCF proteins appear to be substantially grouped together in a cluster (Figure S5A), reflecting that they have undergone remarkable conservation during evolution. Further, the STRING website offers us an annotation prediction of the GO function of this protein network, demonstrating the close correlation between the EsTCP protein and the regulation of branch structure morphogenesis (Figure S5B). This outcome is congruent with what we expected in the *TCP* gene promoter region of Siberian wildrye, which is closely connected to meristem elements, showing that *TCP* transcription factors might possess a vital function in the tillering process of Siberian wildrye.

## 3. Discussion

### 3.1. TCP Genes on St and H Subgenomes of Siberian Wildrye

This study led to the identification of a total of 54 *EsTCP* genes. Apart from the eight genes that were not scaffolded (*EsPCF6*, *EsPCF11*, *EsPCF17*, *EsPCF21*, *EsPCF22*, *EsCYC6*, *EsCYC7*, *EsCYC8*), the remaining *TCP* genes were distributed in two subgenomes (Table S1). There are five chromosomes in the H genome containing *TCP* genes, while seven chromosomes in the St genome are distributed (Figure 1). Moreover, 26 *TCP* genes were discerned in the St genome, which conferred definite advantages compared with the 20 *TCP* genes in the H genome. In addition to the advantages in quantity and proportion, we observed that *EsPCF10* and *EsPCF16* (Table S1, Figure 7 and Figure S3), two genes exhibiting significant relative expression advantages under the five stress treatments, are located in the St subgenome. Furthermore, by thoroughly examining the promoter regions of these two genes (Table S4, Figure 6), we noticed that in addition to responsive elements of auxin, abscisic acid, gibberellin, salicylic acid, and other hormones, there were also responsive elements to low temperature stress and drought induction. Additionally, *cis*-acting elements linked to meristem expression show up in these areas. To manage gene expression, they deftly combine signals from many tissues, developmental stages, and environmental influences. We hypothesize that the *EsPCF10* and *EsPCF16* genes in this study hold an advantage precisely because the St genome exhibits a strong dominance.

Siberian wildrye is an allotetraploid, which is a hybrid offspring produced by doubling the genome of two different diploid species. After hybridization, the subgenomes of allopolyploids often evolve asymmetrically, and one subgenome plays a leading role, which will retain more genes, have higher gene expression, and undergo more evolutionary selection and other events. This process is called biased grading or is understood as subgenomic dominance [47,48]. Owing to a succession of strong dominant genes in the St genome, the morphological traits of diverse allopolyploid combinations of the St genome, such as the *Elymus* (StH) and the *Roegneria* (StY), are converging [49]. Genomic dominance might be one of the reasons accounting for the evolutionary advantage of allopolyploid species [50]. The present work offers indirect theoretical support for the subgenomic and evolutionary adaptive benefits of the St subgenome in Siberian wildrye by examining the *cis*-acting elements and expression profiles of the *EsPCF10* and *EsPCF16* genes (Table S4, Figure 6 and Figure 7). Genomic dominance determines the development potential of new polyploid crops, as well as whether St genomic dominance is strengthened further, we can leverage Siberian wildrye and broaden genetic diversity thanks to dominant genes. Through comparative genomics, the researchers concluded that the H subgenome in “Chuancao No.2” Siberian wildrye was close to that in barley [22]. Our examination of a multi-species phylogenetic tree also shows that the distance between several TCP proteins of Siberian wildrye and barley is relatively near (Figure 2), implying that Siberian wildrye and barley may have an intimate genetic connection. Even so, it is still uncertain which two species contribute to the St and H subgenomes in Siberian wildrye, and more exploration must be conducted to better comprehend the differences between the St and H subgenomes, as well as the essential dominance of the St genome.

### 3.2. TCP Gene Duplication Event in Siberian Wildrye

Gene duplication is the major driving force behind evolution and one method for expanding gene families. Here, *EsPCF2* and *EsPCF3* are located at the same position on the H05 chromosome (Figure 1), and their nucleotide sequences are exactly the same. These two genes are considered as tandem repeats. *EsCYC9*, *EsCYC10*, *EsCYC13*, *EsCYC14*, and *EsCYC15* are closely aligned on the S05 chromosome at similar locations. The sequences are highly similar but not completely identical. Combined with multiple sequence alignment, conservative domain analysis, conservative motif analysis, etc. (Figure 3), it was found that *EsCYC13*, *EsCYC14*, and *EsCYC15* form a gene cluster with similar sequences and similar functions. These three genes are likewise tandem repeats of one another. However, there are subtle variances in the physicochemical properties among the three genes. In theory, two gene copies are created during gene replication, allowing one or both genes to evolve under less strict selection and, occasionally, to acquire novel roles that aid in adaptation [51]. It is possible that the new traits of the species during the evolution of the Siberian wildrye were influenced by the small variations in the physical and chemical characteristics of the three tandem repeat genes, *EsCYC13*, *EsCYC14*, and *EsCYC15*. Via collinearity analysis, we discerned 29 pairs of genes possessing repetitive fragments, encompassing two types of *TCP* genes (Table S2). The discovery that both Class I and Class II *TCP* transcription factors are ascertained to have multiple duplications within the same gene demonstrates the importance of gene replication for the formation of gene families. The gene is conservative in its evolutionary process, as evidenced by the fact that Ka is less than Ks. In most cases, the selection of the *TCP* gene in Siberian wildrye eliminated the harmful mutation and kept the protein unchanged; that is, the purification selection was carried out. Furthermore, 29 duplicate gene pairs contributed to the evolutionary expansion of the *TCP* gene family in Siberian wildrye. The interspecific collinearity analysis revealed that the greatest collinearity was between wheat and Siberian wildrye, followed by rice, barley, Goat grass, and Purle Falsebrome (Figure 4B, Table S3). This fragment replication, which uses another type of gene replication, revealed further details about the genetic link between Siberian wildrye and wheat. Indeed, several wheat grasses (Triticeae: Poaceae) share the St genome [52], which could lead to evolutionary conservation within the common species. In our research, we did a thorough investigation of functional similarities within the *TCP* gene family and discovered that *EsPCF24* and *EsPCF27* show resilient, functional conservation in plant stress response and developmental control (Figure 7, Figure 8, and Figure S3). It is the gene replication event in *EsTCPs* that helps us understand the complexity and diversity of the Siberian wildrye genome, provides thinking for the mining and utilization of important genes, and also promotes the research of other gene families or species, as well as the value of subsequent production and application.

### 3.3. Expression Specificity of TCP Gene in Five Stress Treatments and Different Tissues

Abiotic stress has the capacity to influence plant growth, development, quality, and, ultimately, yield. *TCP* transcription factors are widely involved in the regulation process of plant life and act as key regulators of internal and external signal responses by recruiting other proteins and regulating hormone signal pathways [17,53]. Consequently, it is of crucial interest to examine the latent activities of the *TCP* gene under varied abiotic stress conditions. When we predicted the *cis*-acting elements in the *EsTCPs* promoters, we found that the vast majority of these genes are responsive to stressors like drought and low temperatures and are inducible by plant hormones such as gibberellin, auxin, abscisic acid, and methyl jasmonate. In these results, ABA treatment inhibited 10 *EsTCPs* in roots at all time points (Figure 7). Mutual inhibition between the activity of Class I *TCP* and the ABA signaling pathway is expected because ABA inhibits the processes that *TCP* facilitates, such as plant growth, reproduction, cell division, and elongation. Based on this, the researchers found that [54] *MdTCP46* expression was suppressed in apples during ABA and drought conditions, while overexpression of *MdTCP46* resulted in lower sensitivity to ABA and resilience to drought stress. The distinction is that transgenic Arabidopsis and transgenic rice plants have decreased ABA sensitivity due to the *TCP10* gene found in Moso bamboo (*Phyllostachys edulis*). Through a thorough examination of phenotypic characteristics and stress-related physiological indicators, it was discovered that the overexpression of *PeTCP10* in Arabidopsis facilitated stomatal closure when subjected to ABA treatment. Under drought stress conditions, no notable variation in ABA accumulation was noted between the transgenic Arabidopsis and its wild-type counterpart. Additionally, *PeTCP10* was demonstrated to impede lateral root growth via a MeJA-mediated pathway. In this scenario, *PeTCP10* may work as a positive regulator of plant drought tolerance via ABA-dependent signaling systems, while acting as well as a negative regulator of lateral root growth via MeJA-mediated pathways [45]. Based on the experimental evidence, we postulate that the ABA signaling pathway may play a regulatory role in the root development of Siberian wildrye, potentially through the suppression of specific *TCP* gene expression, thereby modulating root growth and development. Furthermore, it has long been shown in Arabidopsis that Class I and Class II *TCP* proteins regulate leaf growth through the jasmonic acid signaling pathway [53]. Researchers reported that Arabidopsis plants exhibit a root hair shortening phenotype when the *GrTCP11* gene in cotton is overexpressed [55]. This discovery indicates that the *TCP11* gene limits root hair elongation by controlling the jasmonic acid (JA) signaling pathway. However, our research has demonstrated that the *TCP* gene family exhibits a complex regulatory response to methyl jasmonate (MeJA). In leaves subjected to methyl jasmonate (MeJA), the *EsTCP* gene’s expression was also drastically decreased (Figure S3). Interestingly, the *EsPCF5* gene was significantly upregulated in the root and displayed broad tissue expression characteristics following MeJA stress. According to promoter *cis*-acting element analysis, *EsPCF5* contains four methyl jasmonate response elements (Table S4), which possibly be the structural basis for its response to MeJA stress. On the other hand, the absence of typical methyl jasmonic acid response elements in the promoter region of the *DgTCP16* gene in orchardgrass is closely linked to the significant downregulation of this gene upon MeJA treatment [17]. These results imply that the regulatory network between the methyl jasmonate and *EsTCP* gene family may be much more intricate than the straightforward negative regulatory relationship. It may involve a variety of regulatory mechanisms, including post-transcriptional regulation, tissue-specific expression, and a variety of *cis*-acting elements. Similarly, when PEG is used to simulate drought stress, the expression of genes in roots is very little compared with that in leaves, which may also be related to the stress mode of applying drugs to roots in our work, further amplifying the influence of stress treatment on gene expression. The results demonstrated that RNA sequencing under PEG-6000-induced drought stress revealed distinct gene expression profiles between drought-tolerant and drought-sensitive leaves of Siberian wildrye [56]. It was observed that certain differentially expressed genes in tolerant plants exhibited more rapid induction or inhibition. In addition, the *EsSnRK2*, *EsLRK10*, and *EsCIPK5* genes may regulate stomatal closure via the abscisic acid signaling pathway, providing a novel molecular foundation for elucidating the mechanisms underlying drought stress responses in Siberian wildrye. Then, it is plausible to hypothesize that *EsTCP* may play a role in regulating stomatal closure via the abscisic acid signaling pathway. More generally, under the condition of low temperature, the expression of each gene also showed an obvious inhibitory effect. But we can see that the *EsPCF10* gene is less affected by low temperature stress (Figure 7). Hence, we have effectively predicted the existence of several hormone response elements in the promoter region of this gene, such as the precise binding sites of gibberellin (GA), auxin, abscisic acid (ABA), and other hormones. This discovery implies that this gene’s expression may be cooperatively controlled by several hormone signaling pathways, since contributing to plant development and growth as well as stress response.

Molecular biology studies have demonstrated that the promoter region of the *AsTCP* gene family also contains a rich diversity of *cis*-acting elements. Through systematic analysis of promoter sequences, researchers identified 19 distinct types of hormone response elements, with ABRE being the most prominent, comprising 21% of all hormone response elements [57]. Although this study did not further explore the effect of exogenous ABA treatment on *AsTCP* expression patterns, the distribution characteristics of promoter elements strongly suggest that the *AsTCP* transcription factor family may be deeply involved in abiotic stress response networks in oats and play an important regulatory role in plant hormone signal transduction pathways. Plant hormone response elements have been proven to be highly conserved across a wide range of species. Using abscisic acid (ABA), auxin (IAA), and methyl jasmonate (MeJA) as examples, their respective response elements—ABRE (ABA response element), AuxRE (Auxin response element), and JARE (jasmonic acid response element)—have been further identified in Arabidopsis, a model plant, and rice, an important food crop [58,59,60]. The researchers performed a comprehensive analysis of the *AtTCP8* gene targets in Arabidopsis, identifying *TCP8*-bound gene promoters and differentially expressed genes in *tcp8* mutants. These datasets exhibited significant enrichment in components related to various plant hormone signaling pathways, including brassinosteroids (BRs), auxin, and jasmonic acid [61]. And in *Platycodon grandiflorus*, MeJA response elements were found to be engaged in MEJA-mediated gene expression regulation networks. Further research found that when subjected to low temperatures and MeJA therapy, 10 members of the *PgbZIP* gene family demonstrated substantial expression responses [62]. This occurrence is directly related to the existence of *cis*-regulatory elements, which emphasizes their importance. Furthermore, these highly conserved *cis*-acting elements are present in many phylogenetically distinct plant species. This occurrence not only verifies the functional conservation of the *TCP* gene family in the plant hormone signal transduction pathway from an evolutionary standpoint, but it also illustrates a potential critical regulatory role in the plant’s response to abiotic stress. On top of that, the *EsPCF10* gene showed significant expression changes under both low-temperature stress and exogenous growth hormone (IAA) treatment, highlighting its critical role in integrating hormone signaling and abiotic stress response and implying that it may be involved in plant adaptive regulation to various environmental stresses with complex regulatory networks. Significantly, Class I *EsTCP (PCF)* genes have dual functional properties: they exhibit broad tissue-specific expression profiles while sustaining high transcriptional activity under a variety of stress settings. Indeed, these transcription factors serve critical roles in regulating plant morphogenesis and development, especially via altering hormone biosynthesis pathways and reprogramming stress-responsive signaling networks [63]. Also, they manage the processes of cell elongation and the cell cycle [10]. These observations indicate that Class I *EsTCPs* may play different regulatory roles in different developmental stages and may have certain advantages in coping with abiotic stress (Figure 7, Figure 8, and Figure S3). Our findings indicate that several *EsTCP* genes may have powerful adaptive and multifunctional regulation capacities in response to abiotic stress. *EsPCF10* and *EsPCF16* genes (Figure 7 and Figure S3), in particular, demonstrated significant expression characteristics under various stress conditions and were closely related to hormone signaling pathways, making them prime candidates for improving crop stress resistance and agricultural productivity in plateau areas. The work we performed was the first to thoroughly examine the expression patterns of the *TCP* gene family in Siberian wildrye under varied abiotic stress conditions. Compared to earlier studies, which mostly focused on the limitations of single environmental conditions such as salt stress and drought stress, our work made an important advance in experimental design: The research was expanded to include plant hormone control, with a focus on the regulatory mechanisms of major plant hormones such as abscisic acid (ABA), growth hormone (IAA), and methyl jasmonate (MeJA), as well as novel models of low temperature and drought stress. Employing this comprehensive multi-dimensional experimental framework, we systematically elucidated the expression regulation patterns of the *TCP* gene family in Siberian wildrye under diverse abiotic stress conditions. Our work not only addresses a critical knowledge gap in understanding the *TCP* gene expression profiles of Siberian wildrye under combined stress conditions but also establishes a robust experimental foundation and presents novel conceptual insights for deciphering the intricate molecular regulatory networks mediated by *TCP* transcription factors during plant stress responses.

### 3.4. Important Genes Regulating Tillering Among TCP Transcription Factors

*TCP*s from the Class II *TB1/CYC* clade are principally engaged in the regulation of axillary meristem development, namely the generation of lateral branches or flowers. It has been attested in gramineous crops such as maize, wheat, barley, and switchgrass (*Panicum virgatum* L.) that such transcription factors modulate tillering and contribute to the enhancement of biomass [12,64,65,66,67]. In Siberian wildrye, the group *TB1/CYC* includes *EsCYC1*-*EsCYC15*, with a total of 15 genes. Blast comparison with the amino acid sequence of maize TB1 protein (NP_001369512.1) showed that the top three proteins were EsCYC5, EsCYC7, and EsCYC6 (Table S6, Evalue: 1 × 10^−5^), in which EsCYC5 was located on chromosome H06. EsCYC6 and EsCYC7 are unplaced scaffolds, but the sequence and structure of these two genes are completely identical, which may be tandem repeat genes neglected in genome assembly. The subcellular localization predictions confirm that all three genes are localized within the nucleus (Table S1). Additionally, the intra-species evolution analysis and protein-conserved motif analysis uncover that they share remarkably similar structures (Figure 3B), hinting at a high degree of functional conservation among these three genes. In addition, the presence of meristem expression-associated functional elements was further assumed by the examination of *cis*-acting elements in the promoter regions of these three genes. As seen in Figure S2 and described in Table S4, these components might operate as regulators in the Siberian wildrye tillering process. Although we have identified potential meristem-related regulatory components in the promoter regions of *EsTCP* genes, their functional roles remain insufficiently verified. It is, therefore, critical to investigate how these components specifically regulate *EsTCP* gene expression in meristem tissues and their involvement in tillering control. Previous studies have demonstrated the importance of *TCP* family genes in regulating axillary bud development and tiller formation. For instance, in switchgrass, manipulation of *PvTB1* expression through transgenic approaches significantly altered tiller number, stem height, stem diameter, and biomass yield, highlighting its role in axillary bud regulation [67]. Similarly, in Arabidopsis, *BRANCHED1* (*BRC1*) and its homologs act as key local regulators of bud activity, where their expression in axillary buds promotes growth arrest, and *BRC1* mutants exhibit excessive branching due to uncontrolled bud activity [68]. *TILLER ANGLE CONTROL 8* (*TAC8*) has been identified as a nucleus-localized transcription factor that is mostly expressed in the tiller base region of rice [69]. A molecular study revealed that *TAC8* encodes a transcriptional activator from the *TCP* family. At the same time, functional investigations revealed that this regulatory protein improves rice tiller angle by directing cell elongation processes and modulating endogenous auxin homeostasis. In addition, a transcriptome profiling (RNA-seq) investigation discovered that *TAC8* is engaged in two essential physiological pathways in rice: photoperiodic responses and abiotic stress adaption mechanisms. However, the precise role and molecular mechanisms of *TCP* genes in Siberian wildrye remain largely unexplored. Specifically, it is unclear whether *TCP* genes regulate tillering through hormone signaling pathways (e.g., auxin or abscisic acid) or how their expression patterns vary under different environmental conditions. Furthermore, the interaction network between *TCP* genes and other tiller-related genes, such as *TB1*, requires further elucidation. Future studies should employ functional validation experiments, including gene knockout or overexpression, combined with transcriptomic and phenotypic analyses, to systematically dissect the role of *TCP* genes in tillering regulation and their potential for improving plant architecture and yield in Siberian wildrye.

Class I and II *TCP* genes probably perform an essential function in regulation for Gramineae plants like Siberian wildrye throughout critical growth and development stages, including emergence, tillering, jointing, and booting, among others. These genes exert a profound influence on plant growth and development by precisely regulating cell division, differentiation, and organ morphogenesis. As an important agronomic trait that determines forage biomass, the molecular regulation mechanism of tillering has always been the focus and difficulty of herbage genetic breeding research. Our research indicates that the *TCP* transcription factor family holds immense promise for controlling tillering, optimizing plant architecture, and bolstering plants’ resilience to environmental stressors. Consequently, it emerges as a pivotal molecular tool for enhancing plant traits, providing the theoretical foundation and technical support essential for cultivating high-yielding and stress-tolerant cultivars.

## 4. Materials and Methods

### 4.1. Identification of TCP Gene Family Members in Siberian Wildrye

The Hidden Markov Model (HMM) file of *TCP* transcription factors (PF03634) was retrieved from the Pfam database [23]. Subsequently, we utilized the HMMER 3.0 software to conduct a thorough search for *TCP* family members within the protein sequence dataset of the Siberian wildrye genome, as referenced in [22,24]. To seek out fresh *TCP* family members, we conducted a BLASTP (https://blast.ncbi.nlm.nih.gov/Blast.cgi?PROGRAM=blastp&PAGE_TYPE=BlastSearch&LINK_LOC=blasthome, accessed on 18 February 2025) search [27] on the Siberian wildrye genome using TCP protein sequences from Arabidopsis and rice (e-value ≤ 1 × 10^−5^). Overlapping genes are removed with a CDD database search to evaluate the presence of TCP domain structure in all family proteins [25]. Then, the Expasy program (https://web.expasy.org/compute_pi/, accessed on 18 February 2025) [70] was used to predict each protein (kDa) molecular weight and isoelectric point (PI). The subcellular localization can be forecasted through the use of WoLF PSORT (https://wolfpsort.hgc.jp/, accessed on 18 February 2025) [71]. The hydrophilicity and hydrophobicity of proteins were evaluated using Expasy software (https://web.expasy.org/compute_pi/, accessed on 18 February 2025) [70]. TBtools-II v2.152 [72] was utilized to precisely map and display all *TCP* genes along the chromosome of Siberian wildrye.

### 4.2. Phylogenetic Analysis of EsTCPs

We have performed the following actions in order to investigate the evolutionary relationships between the *TCP* families of these seven plants. Firstly, the TCP protein sequences of Goat grass, Arabidopsis, Purple Falsebrome, barley, rice, and wheat were sourced from the plant transcription factor database (https://planttfdb.gao-lab.org/family.php?fam=TCP, accessed on 18 February 2025). Subsequently, these TCP protein sequences, along with those of Siberian wildrye, underwent a multiple sequence alignment analysis utilizing the ClustaW software (https://www.ebi.ac.uk/jdispatcher/msa/clustalo?outfmt=fa, accessed on 18 February 2025) [28]. Via the Clustal online tool, we conducted multi-sequence alignment and divided the *TCP* gene family into three groups in accordance with their alignment outcomes and conserved domains. The evolutionary tree was created using MEGA 11 software’s maximum likelihood approach [73]. 

### 4.3. Examination of the Structural Features, Conserved Motifs, and Cis-Acting Regulatory Elements Within EsTCPs

We discovered 10 distinct motifs in the EsTCP protein by using the MEME software (https://meme-suite.org/meme/tools/meme, accessed on 18 February 2025) [32]. TBtools-II v2.152 was used to help draw and modify the gene structure, motifs, and phylogenetic tree [72]. It is assumed that the promoter sequence is located at the upstream 1.5 kb of each *EsTCP* gene transcriptional initiation site, and the genomic sequence of Siberian Wildrye is extracted according to the common feature format (GFF3) file. *Cis*-acting elements were predicted on PlantCARE (https://bioinformatics.psb.ugent.be/webtools/plantcare/html/, accessed on 18 February 2025) [38], and the *cis*-acting elements of abiotic and biotic stress, phytohormone responsive, and plant growth and development were counted. Using Motif Enrichment (AME) function analysis (https://meme-suite.org/meme/doc/ame.html, accessed on 18 February 2025) in the MEME program [32], enrichment analysis was carried out to determine the regulatory elements in the set of promoter sequences of all genes. A group of randomly interrupted promoters served as controls. The motifs with a *p*-value less than 0.05 were considered to be significant enrichment motifs by the corrected Fisher test.

### 4.4. miR319 Target Site Prediction of EsTCPs

With the aim of predicting the target of miR319, *EsTCPs’* whole nucleotide sequences were examined using the psRNATarget web application [74]. The maximum expected value cutoff is reached at five. Through sequence comparison analysis, the similarity between the predicted target gene and the miR319 sequence was visualized utilizing Jalview 2.11.1.4 software [75].

### 4.5. Analysis of TCP Gene Replication and Collinearity

We employed BLAST ALL to hunt for gene duplications among the identified *TCP* members. The length of the comparable sequence exceeds 75% of the longer gene, and the similarity of the alignment area surpasses 75%, thereby serving as the principal criterion for potential repetitive genes [76]. We utilized KaKs Calculator 3.0 to determine the ratio of nonsynonymous substitutions (Ka) and synonymous substitutions (Ks) within the *EsTCP* gene that originated from a gene duplication event [77]. To ascertain the colinearity of the *TCP* genes obtained from Siberian wildrye, Arabidopsis, and gramineous species of Goat grass, Purle Falsebrome, barley, rice, and wheat, a colinearity analysis diagram was fabricated with the assistance of the MCScanX [34] in TBtools- II v2.152 software. Moreover, the gene replication events in *EsTCPs* were also visualized with the aid of TBtools-II [72], which directly disclosed the orthologous genes within the Siberian wildrye.

### 4.6. Forecast Analysis of the Three-Dimensional Structure and Interaction Network for the EsTCP Protein

The TCP protein sequences of Siberian wildrye were submitted to the SWISS-MODEL (https://swissmodel.expasy.org/interactive, accessed on 18 February 2025) online website for homology modeling [39]. Moreover, the global model quality estimation (GMQE) and QMEAN values are utilized to evaluate the quality of the three-dimensional model. The GMQE score ranges from 0 to 1, with a higher score indicating a more reliable model. The QMEAN score ranges from 0 to −4, with a model having a score closer to 0 being of higher quality. Subsequently, the corresponding PDB files of the predicted reliable models were submitted to the SAVES v6.1 server for assessment. This server encompasses five commonly used detections: PROCHECK, WHATCHECK, ERRAT, Verify-3D, and PROVE [40,41,42,43,44]. If three of them indicate validation, it implies that the model is usable.

To predict the interactions among the TCP proteins of Siberian wildrye, the sequence of the EsTCP protein was submitted to the STRING database (https://cn.string-db.org/cgi/input?sessionId=bFSnR2S3L0Ea&input_page_show_search=on, accessed on 18 February 2025) [46]. Using wheat as the reference species with default advanced settings, we identified 29 sequences predicted to be homologous proteins of wheat. By leveraging the wheat genome data as a reference, we successfully constructed the protein interaction network of EsTCP.

### 4.7. Tissue Collection of Siberian Wildrye and Quantitative Real-Time PCR (RT-qPCR) Analysis of EsTCPs

The vigorous “Chuancao No. 2” Siberian wildrye seed grows in quartz sand and greenhouse. Seven days thereafter, the germinated seedlings were transferred to the modified Hogland nutrient solution. Seedlings were treated with stress when they were growing to a mostly three-leaf stage, about 14 to 21 days after germination. The roots were treated with hormones by dissolving abscisic acid (ABA), auxin (IAA), and methyl jasmonate (MeJA) of 100 mM concentration in the culture medium. A 20% concentration of polyethylene glycol 6000 (PEG) solution was employed to simulate drought stress. When it comes to low-temperature stress, the temperature is maintained at 4 °C, with 14 h of light and 10 h of darkness on a daily basis. The young leaves and roots of plants were collected at 0 h, 3 h, 6 h, 12 h, 24 h, and 48 h after each stress, and each sample was biologically repeated three times. Seedlings that were not subjected to stress treatment were grown continually until they reached the booting stage, at which point new roots, stems, leaves, and ears were harvested from the Siberian wildrye. Then, the samples were collected for RNA extraction and stored at a temperature of −80 °C.

In compliance with the kit’s instructions, the total RNA of the sample was extracted by using the plant RNA extraction kit V1.5 (BIOFIT, Chengdu, China). The reverse transcription kit (Abclonal, Wuhan, China) was used to create cDNA. The RT-qPCR analysis was carried out by means of the SYBR Green dye method, and reactions were conducted on the CXF96 Connect™ Real-Time System (Bio-Rad, Singapore). Based on the phylogenetic analysis of Siberian wildrye’s TCP protein, we picked 10 *TCP* genes from various branches of the evolutionary tree for Quantitative Real-time *PCR*. To make sure the genes chosen were representative, we purposefully omitted genes with high similarity within smaller clades during the selection process. When determining the expression levels of the 10 *EsTCP* genes, the *PP2A* gene [78] is used as an internal reference gene. The relative expression levels were computed through method 2^−ΔΔCt^ [79], and SPSS 27 and Origin 2024 software was used for detailed significance analysis and intuitive data display processing (*p*<0.05). In addition, Supplementary Table S7 lists the primers used in our work for the *EsTCP* gene, which was produced using Primer Premier 5.0 [80].

## 5. Conclusions

As described, 54 *EsTCP* genes were found and categorized into two subclasses, which were then further separated into three groups (Class II *TCP* transcription factor has two branches, 12 *CIN*-type and 15 *TB1/CYC*-type). The majority of genes’ structures and motifs are highly stable throughout evolution, but their activities may alter. *EsPCF10* and *EsPCF16* expression is relatively robust in the presence of ABA, IAA, MeJA, PEG, and low temperature, indicating that Class I *TCP* genes may have the potential to tolerate diverse abiotic stimuli. Simultaneously, the gene was significantly expressed in Siberian wildrye’s ears, while *EsCIN1* was highly expressed in the leaves. The value involvement of *TCP* transcription factors across the many stages of plant growth and development is based on the tissue-specific expression of the *EsTCP* genes. It is also hypothesized that Class II *TCP* genes contain promoter *cis*-acting regions associated with meristem expression, which may play a role in Siberian wildrye tillering. Furthermore, we projected eight miR319 target genes in *EsTCPs*, and MIR319-TCPs might pose a viable target for improving plant tolerance and yield under abiotic stress. Currently, the EsTCP protein’s function is a mystery, particularly in terms of homologous protein expression in the St and H subgenomes. Further studies ought to be carried out to determine its exact function. Our investigation reveals the possible expansion paths of the *TCP* gene family in Siberian wildrye, laying the groundwork for future functional characterization and application of these TCP proteins. The findings not only help future research into the functional activities of EsTCP proteins, but they further strengthen our understanding of protein-protein interactions and the evolutionary history of Siberian wildrye. Moreover, these findings provide valuable genetic resources for high-yield breeding initiatives targeting Siberian wildrye and kindred *Elymus* species.

## Data Availability

Data are contained within the article and Supplementary Materials.

## Supplementary Materials

The following supporting information can be downloaded at https://www.mdpi.com/article/10.3390/ijms26051925/s1.
