# Supplementary material for: Genome-Wide Exploration and Characterization of the TCP Gene Family’s Expression Patterns in Response to Abiotic Stresses in Siberian Wildrye (Elymus sibiricus L.)
Source: Int J Mol Sci. 2025 Feb 23;26(5):1925. doi: 10.3390/ijms26051925 (PMC11900556; doi:10.3390/ijms26051925)
Supplement: Supplementary file 1 [file ijms-26-01925-s001.zip › Supplementary Figure S3-Relative expression in leaves.pdf]

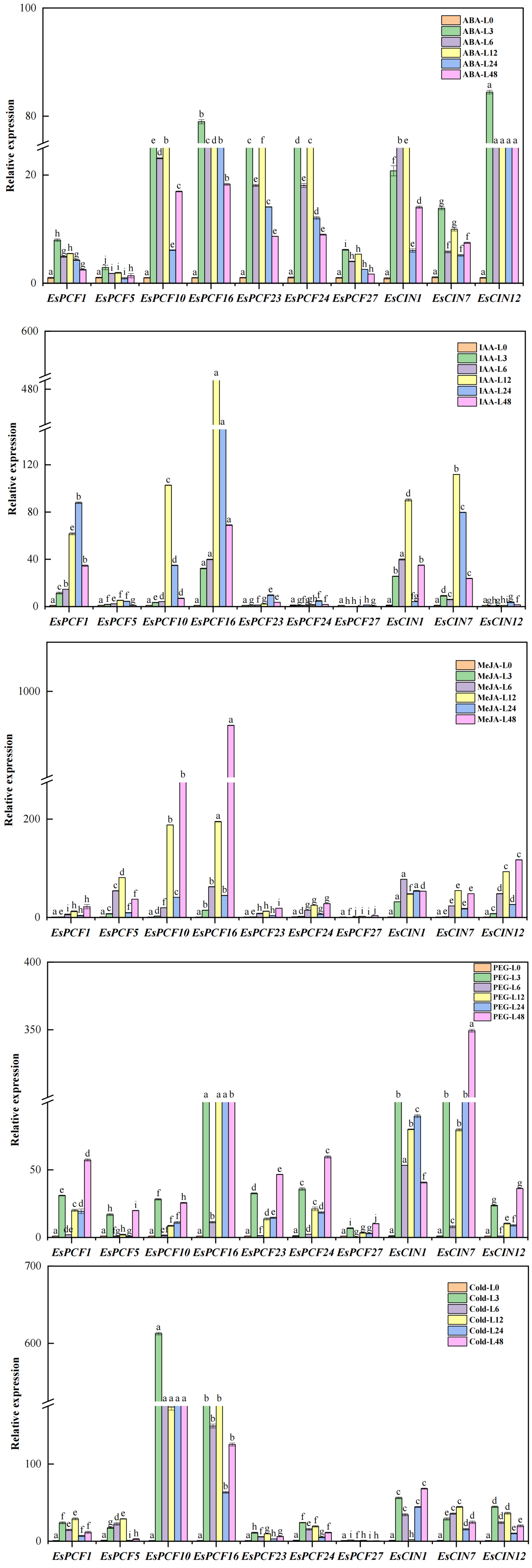

Figure S3. Relative expression of *EsTCP* genes in leaves under five abiotic stresses. Under five distinct stress treatments (ABA, IAA , MeJA, PEG, Cold -4℃), the expression levels of ten genes in Siberian wildrye leaves were assessed at 0, 3, 6, 12, 24, and 48 hours.Significance levels in the figure are denoted by lowercase letters (a-j), with a 95% confidence interval (p<0.05).
