# Supplementary figures and images for: Genome-Wide Exploration and Characterization of the TCP Gene Family’s Expression Patterns in Response to Abiotic Stresses in Siberian Wildrye (Elymus sibiricus L.)
Source: Int J Mol Sci. 2025 Feb 23;26(5):1925. doi: 10.3390/ijms26051925 (PMC11900556; doi:10.3390/ijms26051925)

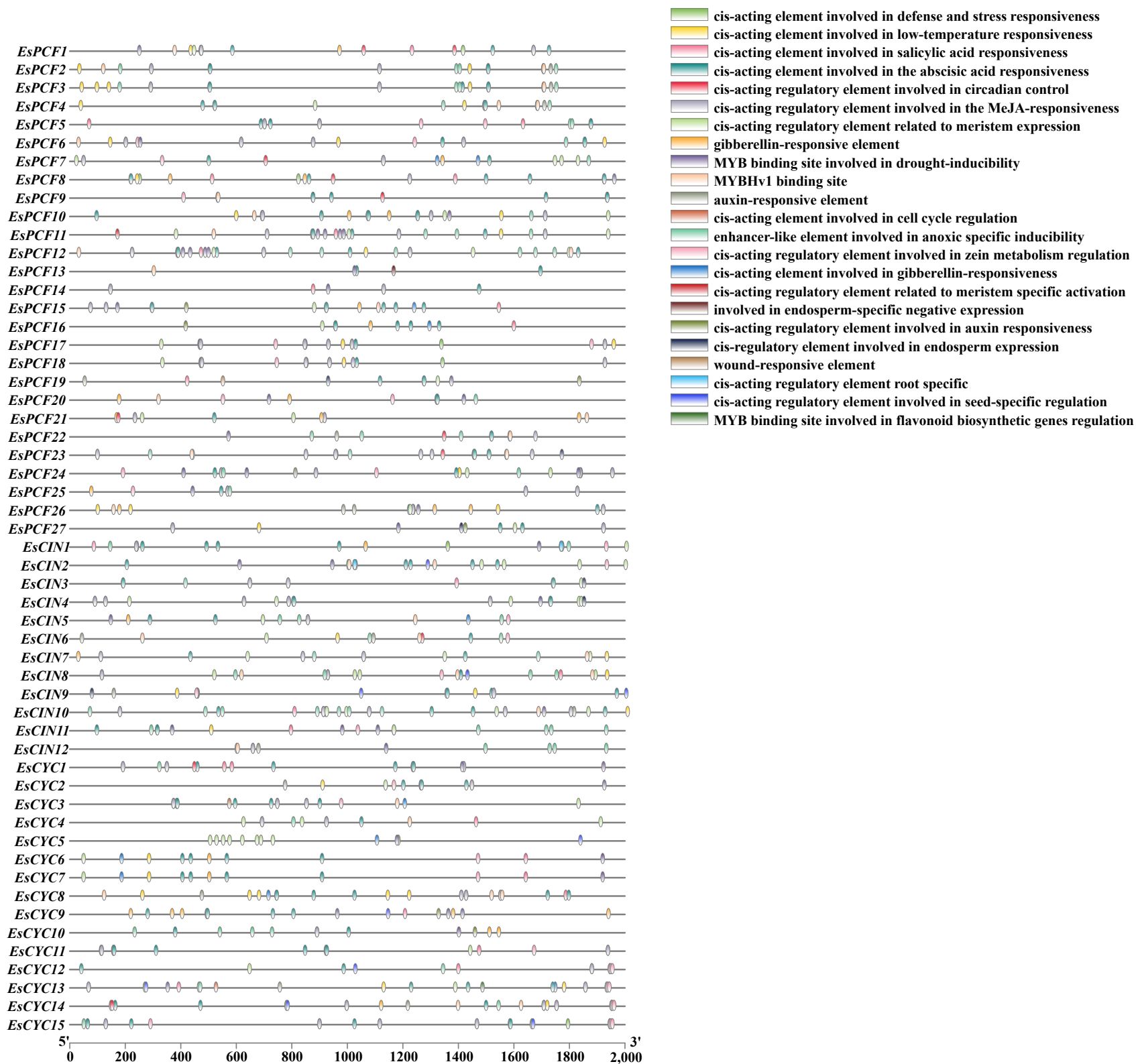

**Figure S2.** *Cis*-acting elements on promoters of *EsTCPs*.

Supplement: Supplementary file 1 [file ijms-26-01925-s001.zip › Supplementary Figure S2-Elements.pdf]
