# Supplementary material for: Genome-Wide Exploration and Characterization of the TCP Gene Family’s Expression Patterns in Response to Abiotic Stresses in Siberian Wildrye (Elymus sibiricus L.)
Source: Int J Mol Sci. 2025 Feb 23;26(5):1925. doi: 10.3390/ijms26051925 (PMC11900556; doi:10.3390/ijms26051925)
Supplement: Supplementary file 1 [file Supplementary Figure S1-Motifs.pdf › Supplementary Figure S1-Motifs.pdf]

Motif 1

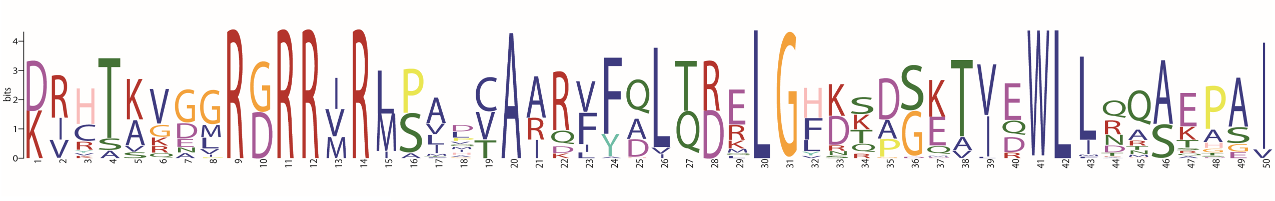

Motif 2

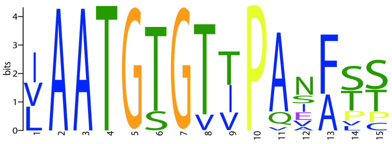

Motif 3

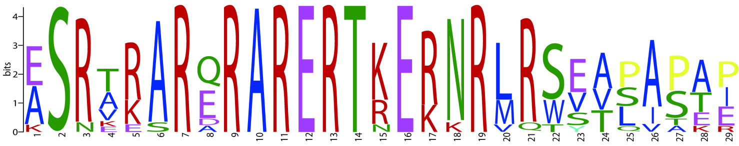

Motif 4

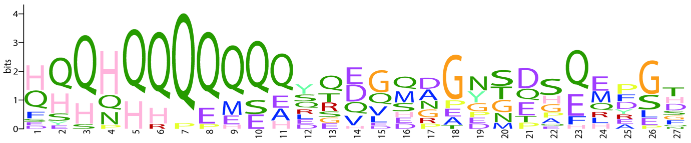

Motif 5

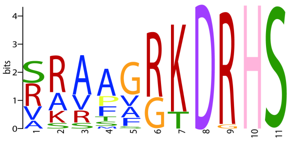

Motif 6

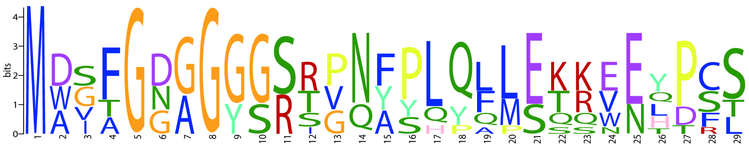

Motif 7

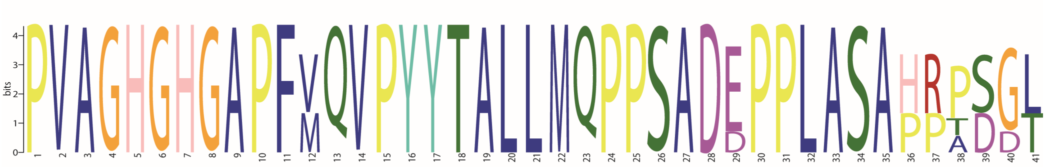

Motif 8

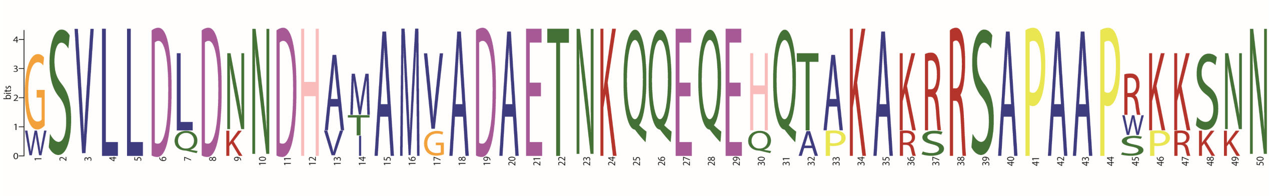

Motif 9

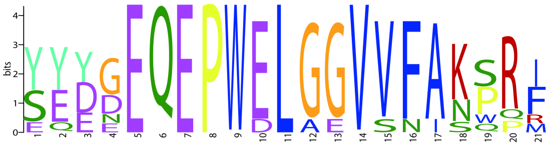

Motif 10

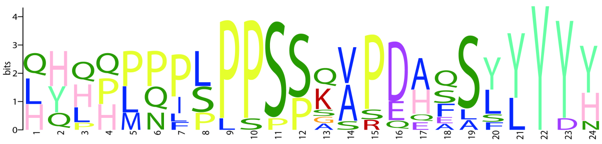

Figure S1. The 10 Motifs of the *EsTCP* genes. X-axis indicates the width of the pattern, while y-axis indicates the frequency of each letter.
